# Use of participatory action research to support Syrian refugee mothers in the resettlement period in Canada: A longitudinal study

**DOI:** 10.1371/journal.pone.0281765

**Published:** 2023-02-21

**Authors:** Joyce O’Mahony, Shahin Kassam, Nancy Clark, Trichia Asbjoern

**Affiliations:** 1 School of Nursing, Thompson Rivers University, Kamloops, British Columbia, Canada; 2 Faculty of Human and Social Development, School of Nursing, University of Victoria, Victoria, British Columbia, Canada; National Center for Geriatrics and Gerontology, JAPAN

## Abstract

Research has shown that refugees in a foreign country often experience physical and mental health challenges upon resettlement (Ahmad et al., 2021; Salam et al., 2022). In Canada, refugee women experience a range of physical and mental barriers, including poor access to interpreter services and transportation, and a lack of accessible childcare, all of which can negatively affect their successful integration (Stirling Cameron et al., 2022). Social factors that support Syrian refugees to settle successfully in Canada have been unexplored systematically. This study examines these factors from the perspectives of Syrian refugee mothers living in the province of British Columbia (BC). Framed by principles of intersectionality and community-based participatory action research (PAR), the study draws on Syrian mothers’ perspectives of social support in early, middle, and later phases of resettlement. A qualitative longitudinal design consisting of a sociodemographic survey, personal diaries, and in-depth interviews was used to gather information. Descriptive data were coded, and theme categories were assigned. Six themes emerged from data analysis: (1) Steps in the Migration Journey; (2) Pathways to Integrated Care; (3) Social Determinants of Refugee Health; (4) COVID-19 Pandemic Impacts and Ongoing Resettlement; (5) Strength-Based Capabilities of Syrian mothers; (6) Peer Research Assistant’s Research (PRAs) Experience. Results from themes 5 and 6 are published separately. Data obtained in this study contribute to the development of support services that are culturally appropriate and accessible to refugee women living in BC. Our objectives are to promote the mental health and improve the quality of life of this female population, and to enable it to access healthcare services and resources in a timely manner.

## Introduction

The world is experiencing the most significant global refugee crisis in history. People are forcibly displaced due to persecution, conflict, violence, or human rights violations [1]. Syrians represent the "largest forcibly displaced population worldwide" [1]. Since the start of the Syrian conflict in 2011, 13.5 million Syrians have fled their homes to escape chemical warfare, massacre, torture, and gendered violence. Approximately 6.7 million Syrians have been internally displaced; 4.5 million Syrians, including 1.9 million children, have requested protection as refugees in the countries of Jordan, Iraq, Lebanon, or Turkey [2]. Evidence suggests that permanently resettled refugees often experience more stable living conditions than those seeking refuge in neighboring countries [3].

Canada has a long history of participating in global humanitarianism. Responding to the Syrian crisis in 2015, the Canadian government partnered with the United Nations Refugee Agency and foreign governments to launch a widescale resettlement initiative [4]. The definition of resettlement is: "the selection and transfer of refugees from a State in which they have sought protection to a third State that has agreed to admit them—as refugees—with permanent residence status" [5]. Approximately 45,000 Syrian refugees (the majority being families), have resettled in Canada through one of three resettlement programs: Government Assisted Refugees (GARs), Privately Sponsored Refugees (PSRs), and the Blended Visa Office Referred Cases (BVORs) Program [6, 7].

Refugees often experience physical and mental health challenges upon resettlement and beyond [8, 9]. Poor language skills, inadequate housing and health services, lower perceived control, and lower perceived social support [8] often burden refugees for several years after resettlement. Although resettled refugees often experience low mental health status, they are less likely to seek supportive services for fear of deportation due to their precarious legal status [9, 10]. Refugee women experience unique gendered challenges in contexts of resettlement. In Canada, challenges can include poor access to transportation, geographical unfamiliarity, a lack of accessible childcare, and unreliable interpreter services—all of which can negatively impact their access to healthcare services [11]. Challenges regarding reproductive, maternal, and child or newborn health [12, 13] can add to this burden when resettled refugee women are separated from their extended family. Social support in the resettlement process can alleviate homesickness, isolation, and mental health challenges experienced by refugee women [11, 14]. Refugee emplacement in British Columbia (BC) has been underexamined in the current literature. Within this gateway province, diverse communities offer affordable housing and cultural connections for refugee families. Framed by principles of community-based participatory action research (PAR), this study examines the perspectives of Syrian refugee mothers living in BC with respect to the social support they received in early, middle, and later phases of resettlement.

The research question was: What factors do Syrian mothers perceive to have been supportive in their integration into BC society?

## Theoretical framework

In participatory action research (PAR), the researcher becomes involved in the living experience of the participants studied. Such personal involvement enables the researcher to generate new knowledge and to act on this knowledge to improve the lives of research participants [15, 16]. In the research presented here, Syrian refugee mothers were positioned as the knowledge holders. These mothers described their experiences in their move to Canada from Syria, enabling the researchers to develop strategies that would improve the social policies that underlie the immigration experience. Our participatory approach enabled us to investigate individual and structural vulnerabilities that affected Syrian mothers who experienced displacement and resettlement in their move to Canada [15, 16]. We used intersectionality as an analytic tool to identify the interrelated factors which shape Syrian refugee mothers’ equitable access to care, social services, and support [17–20]. The importance of utilizing an intersectionality lens generated new knowledge towards equitable outcomes that promote well-being for refugee mothers.

## Methods

To support this longitudinal study, a community advisory board (CAB) was formed to engender community engagement, stakeholder communications, and meetings with the Immigrant Services-Options Community Services Society and the DiverseCity Community Resources Society in the lower mainland of BC. The CAB helped us to design and to implement the study.

### Research design

A qualitative longitudinal design consisting of in-depth interviews and socio-demographic surveys of participants was employed; participants’ personal diaries were examined with permission. To encourage Syrian mothers to impart their diverse experiences during their resettlement in Canada, they were invited to be co-peer researchers [15].

### Recruitment of participants

Forty refugee mother participants were recruited in 2019–2020 via Syrian peer research assistants (PRAs) and social service professionals working with Syrian families. Two recruitment agencies were Options Community Services and DiverseCity Community Resources Society. We also received instrumental support from Mosaic and SUCCESS.

Inclusion criteria: (i) Syrian refugee mothers living in Canada < 5 years, (ii) childbearing age (18–50 years), (iii) current stable mental health (as identified by referral from health and social service professionals). As a point of entry, our research team has existing relationships with key stakeholders working in recruitment areas of the lower mainland of BC.

### Data collection

Data collection included (i) a questionnaire was completed by each Syrian refugee mother to gather sociodemographic characteristics, (ii) each Syrian refugee mother was either interviewed or participated in a focus group, (iii) two focus groups consisting of five and 7 participants were formed as an alternative to an interview, and (iv) each Syrian refugee mother was asked to keep a personal diary (monthly sharing of thoughts, feelings) and to share this diary with investigators via email (smartphone or computer). Through a final group meeting, member checking was done by Syrian mother Peer Research Assistants (PRAs) to discern collective interpretations and directions for action.

Each participant was interviewed for 60–90 minutes using a semi-structured questionnaire written in the participant’s preferred language. The PRAs led the interviews and were supported by a research team member. Interview questions were reviewed for content appropriateness by the PRAs. This interview was modified to match the Syrian mother’s emotional state and her degree of interest in expressing her feelings in certain areas. Open-ended questions were used frequently to encourage participants to talk further about their experiences Most interviews were conducted in English with the support and encouragement of the PRAs. Interviews often involved a mixture of Arabic, Kurdish, and English dialogue. The PRAs provided translation support and confirmed the interview data. As the interviews could potentially uncover sensitive issues that might distress the Syrian women participants, a counselling service was available. Each interview was audiotaped with the participant’s consent and conducted in the participant’s home until March 2020 when the Covid-19 pandemic erupted. During the Covid-19 pandemic, follow-ups took place via telephone and email. Participants’ diary entries were gathered each month by a PRA via email (smartphone or computer) until the close of data collection at 18 months. Diary prompts were open-ended questions asking participants to reflect on thoughts and feelings influencing their well-being.

### Data analysis

Braun and Clarke’s (2006) steps for data analysis guided the thematic analysis. To inform research team members of emergent themes, preliminary analysis of the data was continuous during data collection [21]. Data and field notes were taped and transcribed verbatim following the interviews with Syrian mothers. (i) In the early stages of analysis, transcripts were coded to identify preliminary themes from the data and a list of code categories for organizing incoming data was formulated. Code categories were refined as subsequent data were gathered. (ii) The outcome of this analysis was a set of complicated, interrelated concepts and themes. This process was cyclic as the iterative thinking process occurred over numerous months, including the collection and analysis of interview and focus group data and personal diaries. (iii) Themes and concepts were used to compare within and across transcripts in the data set and across cases. To assign units of meaning to the descriptive data, which included field notes and memos, chunks of varying words, phrases, sentences, or paragraphs, were used to enable the research team members to identify and analyze what was important. The computerized software program QSR NVivo 12 assisted in the analysis and in management of the data.

### Ethical approval

This harmonized study was approved by Thompson Rivers University and the University of Victoria through Research Ethics BC. Informed consent was obtained in Arabic, Kurdish, or English. Participants were duly informed of the procedure for, the risks of, and the benefits of participating, and their right to withdraw anytime during the study.

## Results

Six themes emerged from the data analysis. Four themes (1, 2, 3, 4, above) are reported in this article. Theme 5 (Strength-Based Abilities of Syrian Mothers) and theme 6 (Peer Research Assistant’s Experience) are published separately.

### Theme 1: Steps in the migration journey

Refugees to Canada face multiple risks related to mental health during all phases of the migration journey. Thus, the immigration journey does not end as soon as the immigrant arrives in Canada. Participants described feelings of displacement. They witnessed traumatic events during their journey. Their separation from family, friends and freedom presented a loss of the stability they had known in the home country.

**Premigration.** Refugees to a new country leave livelihoods, jobs, family members, and homes. They often feel uprooted and lost when they reach their destination. Participants were asked about their lives in Syria, before fleeing their country.

Participants described that in Syria they were surrounded by an extended family with built-in caring family members. Most participants were emphatic that: “*In Syria I could go out shopping but its not necessary because*, *like*, *my mother-in-law could go*, *my*, *my brother-in-law could go*, *could do everything*, *my husband could do everything*.*”* [P0034]

Their narratives strongly acknowledged that life was satisfying before the war (the Syrian civil war began in 2011 and is ongoing), even “perfect” as one participant highlighted:

*Before the war for most of the people everything was perfect*. *Extended family was there to help*. *But now in the city there is only the essentials because they don’t want people to stay*. *So*, *in a sense there is no choice to stay*. [P0020]

Many mothers reminisced about Syrian life and the accepted and traditional roles of women:

*But in Syria even you finished your degree you and get married you can’t have the freedom to work as here because the traditions as and her responsibilities to cook taking care of her kids and her husband and sometimes for her family-in-law*, *too*, *prevent her to continue*. [P0026]

*We didn’t work*, *we make so little* (for) *woman work in Syria but when the woman changes her life and come here should the woman work*? *Women in Syria work only as housewife*. *In Syria we don’t think about working*, *because like everything possible*, *like*, *my husband working full time and he gets*, *like*, *very good pay*. [P0032]

As war progressed in Syria a mother lamented about her war related loss: *“My husband died in front of my eyes*, *me and my kids*, *he died during the war*. *We feel very*, *very bad when we*, *think about this …”* [P0017]. Another participant recalled their somewhat ambivalent decision to leave their home:

*I feel not good*, *I feel bad*. *How can I take my kids and go to*, *by boat*? *I hear some news some people died*, *I tell my husband*, *no*, *please*, *I tell him*, *no*, *we can’t*, *we*, *but then we just decided this week we will go at twelve midnight*. [P0019]

One participant bravely unravelled her story of the plight to flee Syria and seek safe refuge with her family. This young mother recounted her plight to leave and cross the Syrian-Jordan border:

*War was at the highest*, *the labor pains started*. *Snipers were spreading on high surfaces and shoot at everything moving … almost midnight my husband and I with my mother-in-law used the window to get out and hide among the trees*. *We walked arm in arm through the rain*, *mud*, *and darkness toward a safe place away from sniper bullets*. *A border roadblock they check papers*. *I started crying and shouting to let us through*. *After we arrived hospital*, *I gave birth two minutes later*. *People think that mother gives life for their child*, *but I think that my daughter is the one who gave me life*. [P0020]

This woman’s account is an example of how premigration experiences can be framed by simultaneous advantage and disadvantage, through intersections of social support, extended networks, gendered roles, care-taking responsibilities, but also through extreme loss and trauma. Many Syrian mothers longed for their premigration home. The social advantages of Syrian mothers were dependent upon the social context in Canada where perceived safety was impacted by thoughts of home and ongoing war in Syria.

**Migration.** In the early (primary) migratory phases, many refugees experienced discrimination from intersecting social, cultural, and economic circumstances [22, 23]. For example, in Jordan, over 660,000 men, women, and children are currently trapped in exile. Approximately 80% of them live outside refugee camps, while 128,000 have found sanctuary in refugee camps such as Za’atari and Azraq [23]. All the participants in this study experienced secondary migration, also known as mixed movements, which involves a transit through one or more countries during a forced migration journey [24]. Upon leaving Syria, most participants landed in Jordan, although a few settled in Egypt, Lebanon, and Turkey. As a Syrian mother made clear: *"No one comes directly from Syria* [to Canada], *everyone came from a secondary migration … but from there to Canada*, *it is a choice*.” [P0020].

Syrian mothers and their spouses reported an inability to find and maintain job security. Many Syrian families had arrived with limited means to cover even basic needs, and those who could at first rely on savings or support from host families were now increasingly in need of help. In Jordan, about four out of five Syrian refugees (close to 80%) were living under the national poverty line even before the COVID-19 pandemic, surviving on about US$3 a day [25].

Syrian mothers expressed several major hindrances that disrupted coping with life in Jordan. Economic instability was a common hindrance. Many of the women’s husbands participated in heavy labour and/or precarious work, and payment for this work was unpredictable. Many of the Syrian mothers felt that these work conditions were discriminatory. They described their own experiences in Jordan as discriminatory; they did not feel like they belonged.

*Because you are a Syrian so you just give us*. *They give the Jordan worker*, *like*, *twenty-five*, *they give the Syrian only five dollars*, *her husband was like that and sometimes they didn’t give him anything and the end of the month they give him just small amount … In Jordan they didn’t give the Syrian workers*, *like*, *the right amount of money*. [P0028]

Several participants noted that it was risky to work in Jordan.

*My husband he can’t work at Jordan never*, *never*, *if the government see my husband work they take my husband and send him back to Syria* [P0019]. [You need] *a license for a job; you don’t got a license you can’t work*, *maybe come back to Syria*, *yeah*, *the license very expensive*, *seven hundred dollars in Jordan*. [P0034]

Birth experiences were particularly difficult in the unfamiliar context. Feelings of displacement with limited or no extended family member support were devastating to many participants. Back home, Syrian mothers were used to having extended family supporting the birthing event. Birth experiences were described as strict, and without consideration of the Syrian’s mother’s emotional wellbeing. One participant elaborated on her labour:

*So*, *like I asked them for help but they didn’t say or do anything*, *so*, *like I was falling down the ground*, *but even though*, *like*, *I didn’t get any help*, *like they didn’t allow my*, *my mum or anyone to go inside with me because that’s a rule*. *There’s a rule in Jordan you can’t*, *like*, *bring anyone with you*. *Even*, *like*, *with my pain*, *and then the problem is*, *like*, *they were saying bad words for me … stop bringing more kids*, *like*, *and that Syrian ladies*, *like*, *have to stop*, *come on*, *go back to your country …* [P0040]

Many of the Syrian mothers reflected on their somber experiences of feeling unwanted and being treated poorly. “*You are from Syria*, *you are not good*, *your country not good*, *you are as a person not good*.” [P0038]

*There were too many difficulties we faced; for example*, *like we lived in a small town*, *like*, *far away from the city … we prefer to stay in the house then go outside and*, *like*, *we heard some people talked to Syrian people like go back to your country and this stuff*, *or you steal our jobs*, *you steal our men*, *and things like this … is what we hear*. [P0040]

This woman’s description highlights systemic discrimination based on national identity which compounded Syrian mothers’ worries about their need to maintain some economic stability. Gendered roles and the responsibilities of mothering prevailed for the Syrian mothers, but the stigma of exclusion further contributed to the traumatic impact of their forced displacement.

#### Postmigration

Postmigration was defined by participants as their arrival in Canada. This second resettlement was a difficult process with an uncertain timeline [26]. The refugee women needed to adjust to the socioeconomic and cultural norms of their settlement community while attempting to learn the official language [27]. Multiple factors can influence refugee women’s mental health and well-being post-migration. Immigration status, gender roles and expectations, ethno-religious identity, language, and socioeconomic status simultaneously challenge and support women’s mental health outcomes [27–29]. Discrimination, poverty, and social exclusion contribute to the development of mood and stress disorders among refugee women. However, other studies show that refugee women often develop strategies to manage their stress and protect their mental health during resettlement [30, 31]. Sub-themes of refugee women’s expectations and challenges to resettlement emerged.

Some participants had mixed views on their expectations of Canada. One Syrian mother clearly noted: “*I’m settled and here now*, *and I don’t think back because I lost everything*, *like*, *there’s nothing left in my country*.” [P0020]. It was said that relocating to Canada was a benefit for their children’s education. One Syrian mother said *“Moving*, *its really hard*. *I will be honest with you but*, *like*, *for your kids*, *their good education*, *like*, *if you look at that side there’s good education for your kids*.*”* [P0033]

Dreams became real for another Syrian mother:

*Sometimes I felt like it* [Canada] *would be close to my culture*. *In Syria we were asking for our rights*, *but here I felt like it wasn’t my right to ask*. *It was a dreaming state to realize now it was my right to ask*. *My kid was watching a movie and asking for a playground in Syria which I knew wouldn’t happen*. *After resettlement I found many of those things I dreamed about before*. *Dreams became reality*. [P0029] Some participants had the opposite experience. Facebook painted a prettier picture of houses than the housing in which refugees were accommodated. Arriving in Canada punctured this balloon: “*We will offer a nice life for us*, *and then when we came … But this is a surprise*, *like … like there’s nothing*, *no job*.” [P0032]. Another participant was exhausted with the resettlement phase: “*I did not expect to find such suffering for my children*, *desperate and sad*. *I am tired of bitterness to live and I hope to go back where I came …*” [P007]. Another participant felt betrayed by the poor housing conditions she found in Canada:

*The first day its very bad*, *uh*, *because I expect something different from what I see*. *Everything change with the people*. *I don’t see these people before*, *and my kids*, *too*, *very sad about this*. *Its*, *uh*, *not clean … a little bit dirty*, *too much mess and*, *um*, *mice*, *there’s mice in my house*. [P0021]

One participant fully expected to settle into a home soon after her arrival in Canada. She naively thought that: “*When I come to Canada my situation is going to be changed … not kind of rich but will own house and not rented … for four years in Canada I still rent a house*.” [P0024]. Another participant expected to live in a bigger, nicer, and cheaper home compared to her past homes. She described her living conditions—with three children in a basement suite with limited daylight—as depressing.

Upon arrival in Canada, refugees faced challenges to resettlement. These included being met by Canadians with negative attitudes, difficulties in finding employment and housing, and the absence of an extended family that would have provided support. One participant recalled:

*Like*, *one morning at five a*.*m*. *we heard someone doing the hammer*. *The following week the manager of this apartment*, *he give us a notice that we made a noise and we have to pay fifty dollar*. *My husband say also we wake up on that first morning*, *that doesn’t mean that I did the hammer*, *that wasn’t me*, *that wasn’t my kids*. *Imagine how*, *like*, *we have two kids and what to do*? [P0002]

Due to neighbor issues, one participant expressed concern for the safety of her children:

*Okay*, *I was in house and the rent*, *the landlord of the house he was very*, *very bad with me*, *with my kids*, *all the time talk bad words with my kids*. *And*, *um*, *I shocked when I think everything good in Canada*, *but when I arrive to Canada*, *I shock all through the year*, *I didn’t know what in English*. [P0017]

Another participant said she felt judged, and she detected a negative vibe toward her because of her religious beliefs:

*Even we are Muslims we have the right to live how we want*. *Sometime my kids fight outside people looking at me*. *I feel that they judge me*. [P0002].

Another participant attributed the negative attitudes she detected to discrimination or hate toward Muslims and requested mutual respect.

*They have a mental problem*, *not mental health*. *Yeah*, *it’s they develop it* [in] *their mind*. *The hate towards Muslims or*, *like*, *it’s a special thing*. *Like*, *as I respect whatever you do*, *whatever you wear*, *wherever you go*, *just respect that for me*. *I don’t ask you for anything else*. [FG-001]

After arriving in Canada, participants described inadequate living conditions and the absence of support or help:

*So*, *like the first days in Canada*, *so*, *it was terrible*, *like I lived in a basement with*, *like*, *the washing machine and this stuff … so we stayed*, *like*, *for a week without any help*, *without support from anyone*, *so*, *like I have to feed my kids; so then after a week I went to ask them*, *so I have to feed my kids*, *like*, *I need the help*, *I have no idea about surroundings*, *like*, *so*, *I ask them for help*. [FG-002]

After the initial temporary housing, participants described having difficulties in finding a more permanent place to stay, and the trouble they had in navigating their new surroundings:

*Yeah*, *the most difficult thing here housing … we found people to help us to find food bank for food*, *to show us the store*, *the Superstore*, *everything*, *but the most difficult*, *the housing*, *because expensive maybe … so expensive*, *if we didn’t have*, *if you didn’t find house to us why you bring us to here to Canada*? *Just we*, *we want to come back to our country*. [P0030]

Consequently, some participants said their children had reached a point where they longed to go back to Jordan:

*One month and twenty days with no food*, *no anything*, *one month and twenty days*, *no anything*, *that’s why we*, *we just*, *uh*, *we feel very bad*. *My kids*, *now they said we want to come back to Jordan*, *we are not happy here*. [P0017]

Challenges to resettlement included longing for and worrying about extended family left behind in Jordan: “*When I came to Canada*, *I feel stressed and I feel sad because my family*, *all my family*, *in Jordan*.” [P0042] “*They live in fear because there are many cases of child abduction*.” [P0019] Some participants pointed out that they had become solely responsible for areas of their lives where they had previously relied on the support of an extended family:

*It’s really hard*, *like*, *thinking about back home*, *and here I have to do everything by myself*, *yes*, *it is really hard*.” [P0028]

*Everything different*, *there’s no family here*, *but*, *… I don’t really go to school when I’m tired and pregnant*. *I don’t learn how to drive*, *yeah*, *everything here I have responsibility*.*”* [P0034]

Participants experienced a wide array of challenges to resettlement. Challenges such as finding employment and suitable housing, while worrying about one’s family left behind, require support in the initial stages. Refugee mothers need support services to be introduced early on to help them navigate the challenges they will meet at the onset of resettlement. If initial challenges are met with support, the years following resettlement will go more smoothly.

### Theme 2: Pathways to integrated care—holistic healthcare needs of Syrian women

Participants were asked about their emotional and physical healthcare experiences. Emotional health, physical health, and access to healthcare emerged as important intersecting healthcare needs. The refugee women relayed mixed experiences in their help-seeking activities, including positive and negative factors that influenced their help-seeking behavior. Participants were divided on whether healthcare services supported their unique situations. Some participants had language difficulties, low energy, and felt stretched thin among personal, social, and financial pressures.

#### Emotional health

According to some Syrian mothers, both emotional health and physical health are integral for performing activities of daily living and for learning new skills in resettlement. Many mothers leaned on their cultural and spiritual practices to address their emotional well-being. Many participants described that it was difficult to be healthy emotionally without a social support network.

For this Syrian mother it was devastating to not have the emotional support of her mother and she was afraid to share the burden of her refugee experiences with her mother:

*… for me in the past*, *when I find anything hard*, *I just call my mother and tell her … but not in Canada*. *In Canada I haven’t told her about anything*, *yeah*, *because*, *like*, *she has lots of hard things and guilty; like*, *there is one of my brothers in the army and its not his choice*, *they force him to go*. *The most difficult thing is I can’t give her more difficulties on her shoulder*, *yeah*, *that’s enough for her*. *So*, *I just get silent*, *I don’t tell them even if I want to tel*l. [34]

After resettlement, participants who left extended family behind were often troubled about family members. This had a strong impact on their mental health and wellbeing. Some of the horrific experiences recounted by participants illuminated why they chose to leave. One participant wrote in her diary:

*With the beginning of October*, *we woke up to hear the news of the martyrdom of my husband’s younger brother*. *It was very unfortunate and sad news*. *The moment of joy that flooded his heart and ours did not complete when we heard the birth of a child for him*. *It was preceded by very sad moments*. *I saw his baby who was only five hours old*, *and he only saw him for a moment*, *and only the sound of his crying was heard from him for once*. *Yes*, *my husband’s younger brother was martyred*. *He was killed and burned after he was shot dead*. [P008]

Such narratives show that worrying and thinking about extended family left behind is unavoidable for refugee women.

One participant described her role and her responsibilities in their new home.

*It was my role to calm him* [husband] *down even with two small kids*, *but I have a big role in my house*. *Sometimes I have a lot of stress because the responsibilities I have*.*”* [P0002]

Another participant acknowledged that the family would sometimes rather go back to where they came from.

*Sometimes I think about returning to my home country because I did not find what I came to Canada looking for*. [P0020]

The lack of extended family was especially unsettling for some participants after they gave birth. Post-birth support for many of these women ended abruptly upon resettlement. The lack of social support from family members that refugee women experience is well documented [3]. Participants explained that in their culture the extended family plays a big role after a newborn is welcomed to the family:

*Ah like being a mum its difficult*, *but in our culture we use the family member to help us*. *But when I arrive*, *I shocked*, *like it was a huge duty on me*. *I had a big responsibility*. *My kids are too close to each other*, *like almost twenty months between them*.” [P0002]

*I was missing my mother and all my family because they are far away from me at this time*. *To share my joy in giving birth to my baby*. *But for the first time they will not be beside me*. [P0009]

This participant described how Syrian mothers are needing to take on the responsibilities of a newborn by themselves which leads to poor outcomes including feeling overwhelmed and isolated. This highlights the important roles that mothers, and mothers-in-law play in the Syrian culture. However, these mothers were not bereft of support; participants referred to their husbands as the most supportive persons in their lives. When participants were asked to whom they would turn for support, participants cited the support of their husbands: “*My husband supported me during that tough time; without his help it could took me longer to talk about my feelings*.” [P0002]. “*Okay*, *so when my husband beside me*, *so I feel I’m the strongest person in the world*.” [P0042]

*I’m social person … lucky I have a lot of friends*, *and the most supportive person in my life is my husband—he is my husband*, *he’s my mum*, *he’s my father*, *he’s my brother*, *he’s everything in my life and he never let me think that I’m losing something—he always make me busy with something to do*, *yes*, *so he’s the most important person*. [P0007]

Another primary challenge that participants shared post-birth were feelings of isolation: “*I’m crying without any reason*. *Also*, *my neighbor who I get to talk usually*, *she will be moving and I will be alone again*. *I do not have anybody to talk to*.” [P0024] “*I have been at home for two weeks*. *I did not see anyone*. *It is very difficult*. *When I don’t see anyone*, *I get depressed*.” [P0006] *“I worry the baby’s coming and nobody*, *no family*, *no mum*, *no sisters … Yeah that’s why I feeling sad*.*”* [P0020]

Another participant preferred to keep her feelings to herself rather than share them with friends:

*I feel for sure I feel isolated because*, *like*, *there’s nobody around me here … And*, *like*, *every day the kids in school and I stay alone*, *nobody come to visit me*, *no friends*, *no one seeing us*. *I was thinking about my city*. *I didn’t spend one single day*, *like*, *alone … in my country I like the time goes really fast turning*, *but here the time is not go at all*, *I spend the whole day alone—I feel for sure isolated*. [P0033]

These participants’ descriptions point to the collectivist culture of many non-Western countries where the integration of extended families, religious groups, and ethnic communities into social support systems seem beneficial for refugee women. Social networks can provide support from women of similar backgrounds and from faith-based groups. Participants conveyed how this support can positively influence the mental health of refugee women. Participants acknowledged that such facilitators supported their emotional security and wellbeing during resettlement. They especially appreciated agencies that had programs to educate newly arrived refugees and families with young children in the early years of assimilation.

*The first day I come home and this program called me please come in this week*. *I am coming here*, *I see many languages here in Canada*. *She tell me if you have any problem or any appointment I can go with you*. *I have a small family*, *I bring my kids everyday*, *come here about one month*. *After that I go to school*, *I see many friends and people to help me and my family*, *no stress*, *start group my language*. *I think Canada is good*, *good for me and my kids*. [FG002]

Religion was identified as a steadfast support. Clearly, religion and spirituality play important roles in the emotional wellbeing of Syrian refugee women. Religion offers social support for many of the difficult hardships faced upon resettlement. All participants expressed that their religious and spiritual practices offered them strength and a way forward to cope with emotional and physical health challenges.

*Because I’m Muslim people*, *you know*, *Muslim people pray and read Quran*, *and that’s very good for me*, *sometimes I just read Quran and always pray*. *… we have five times for our prayer*. *we didn’t know everything about my religion*, *we need to listen every time*, *always*, *to listen many things …* [P0020]

*At this point of my life I felt so stressed … I asked myself what’s the reason for all of this stress … the most important thing*, *like*, *the God told us if you feel you are so far from God or from being understood*, *yeah*. *I believe in that and*, *like*, *the prayers we say*, *just that’s a ritual thing and it has a strong reflection in my life always*, *going through any problem I just say some prayers and I feel its all done*. [P0034]

The communication style and the dress of the refugee mothers were distinct underlying factors of cultural belonging and identity, and were strongly related to the emotional well-being of participants. Traditional dress was voiced as symbolic for cultural and religious identity. One participant expressed gratitude for the support provided by church sponsors who encouraged the women to reflect their true selves and not being afraid to stand out:

*They did a lot for me*, *introduced me to many places—library*, *community center*. *We used to go with them to parks*, *playgrounds*, *to some events*. *They invited us to the cinema*. *It shows me I am an important person*, *with my hijab I feel that I am working with Canadian people and other people are seeing me*. *I am with people who*, *who feel*, *like*, *feel safe*, *who feel like other humans*. *We are together like this and I wished we went outside more*. [P0038]

*I feel bad*, *but after four or five months*, *like*, *I felt*, *I felt like we are here in Canada*, *like*, *I realize that there is many*, *many*, *religions here*, *its multi-culture*, *not everyone is going to like why are you behaving in this way—you have the right to do anything*. *You have the right to live how you are*. [P0002]

Some participants felt that wearing a Hijab inspired negative attitudes and stereotyping among Canadians.

*Maybe they see my hijab*, *or they know we are*, *like*, *I don’t want to say they’re discriminate because I don’t know*, *some people they don’t show it*. *Yeah*, *because of my hijab I think people are looking at me or judging [saying] “she’s Muslim*, *she’s doing this”—they think they have an idea*, *like*, *a story*. [P0031]

Another emerging concern that was related to assimilation and emotional wellbeing was the participants’ style of parenting. Some participants observed a difference in parenting between Syria and Canada:

*When I was another home*, *my neighbor sometimes had my children and sometimes crying*, *they call to police and they say to him*, *me and my husband* [speaking Arabic] *hit*, *hit my kids … the police come to my house and they say you have this problem*, *your kids*, *you hit your kids*?… *kids sometimes when they get together*, *sometimes like hit each other… Yeah no*, *problem here*. [P0025]

*The kids*, *back home or even in Jordan if you tell them don’t do that because I will punch you*, *that’s enough for them to be*, *to have a good behavior*, *but here if you say I will punch you they would tell you and call the police*. [P0025]

Due to differences in parenting between Syria and Canada, one participant worried that Canadian people might believe that she was not taking good care of her children:

*Syrian mums*, *she has experience for the children and but it needs more*, *more like how to deal with her kids here*, *especially in Canada because here some habit I got it from my back country*, *like*, *its not allowed here*, *actually*, *not*, *even leaving him alone not allowed*. *So*, *some mums they don’t accept this situation*, *or can’ t … So parenting skills are different*. [P0026]

*In the beginning it was very difficult for me to get them [children] outside together*. *It was like they’re doing something wrong and the people blame me*. *I was thinking*, *is this … something wrong*? *They are going to*, *like*, *see me*, *what mum you are*, *you’re not taking care of your kids*. *I was scared from this idea*. *But after a while*, *like*, *I discover there’s nobody can charge me*. [P0002]

#### Physical health

When asked about their own health needs, participants often referred to the physical health of their extended family, not their personal health. Participants worried about family in Syria and sending money home.

*She talked about her niece and how she is sick*. *Her brother lives in Syria and the problem that he does not have any money to take care of his daughter*, *so she decided that she wants to send him some*, *but her husband did not agree*, *so she offers that she is not going for shopping this month at all*. *Then she could save some money and help her niece*. [P0017]

*This month has been very tough*: *her brother is very sick*, *in hospital for last couple week—for 10 days in the ICU*. *Got a chest infection*, *has oxygen now and can talk now*, *feeling stressed*. *He’s been here for 13 years—trying to get parents here from Syria to spend time with brother*. [P0002]

For patients with pain, heart, or chronic conditions, social isolation was a recurring theme. Some participants noted how social isolation can exacerbate physical pain:

*My week is just as a day I am sick and the other day I am so glad to hear good news from my children about their school or any thing else so I forget my pain and my illness*. *The rest of the week I stay alone so I remember my pain—my pain increases*. [P0024]

*When the people isolate you from surrounding it is painful and hard to enjoy your life*. *All of this overthinking lead me to isolate from everything*. *Normally I don’t talk to my friends about my feelings*. *I am trying to keep it for myself*. [P0002]

#### Access to healthcare

Factors that facilitated seeking help included formal support and services provided by healthcare providers and other community resource agencies. Participants also acknowledged informal support given by partners, family members, and friends. Many participants were pleased with the care they received, especially from their community health nurse and the follow up care provided post-birth. One participant highly praised the nurses who cared for her during labor and delivery:

*The second baby*, *I birth him here*. *Like it was really difficult for me giving birth*, *but the treatment was really good*, *like*, *every second a nurse came for me*, *try to calm me down*, *like it will be fine*, *it will be okay*. *I felt like even I could not understand what they saying because of English … I could feel it because they are smiling and … calming me down*… [P0040]

With no interpreter services offered, unexpected costs, and long wait times to see a healthcare professional, the healthcare services were challenging to the refugee women.

*When we arrive to Canada the most important*, *like*, *they have to prepare the interpreter for everyone*. *When I got here we all get sick because*, *you know*, *change of the water*, *change of the environment*, *so all the family*, *we went to the doctor*. *There’s no doctor that speaks our own language*. *So how we going to explain what we feel to the doctor*? *And how we can get*, *like*, *the correct medicine*? [P0024]

*My son fell down*, *… the ambulance come to my house*, *okay*. [Paramedics] *want to make me relax*, *“your son is good*, *don’t worry*, *don’t be afraid*.*” When I arrive to the hospital*, *they ask me to pay seven hundred fifty* [dollars].

The refugee woman’s brother stated: *“She came by UN and she covered*.*” The paramedics asked*: *“where is the healthcare card or anything proof*?*”* [P0017]

The refugee women participating in this research spoke of a lack of translator services in the healthcare sector. This is concerning in that overwhelming research has found that trained professional translators are an asset to both the newcomer and the healthcare provider [9, 28, 32].

### Theme 3: Social determinants of refugee health

#### Intersecting dimensions of Syrian women’s health

Syrian mothers’ narratives reveal how resettlement factors and policies interact to influence women’s overall health. We asked the Syrian mothers how the resettlement factors influenced the health of their families. Subthemes comprised language, role of gender, education, housing, and employment.

**Language.** According to all Syrian mothers, the most important thing upon arrival in Canada is to have a language interpreter. Language skills can act as both a barrier to and a facilitator of integration in the resettlement period. One participant noted: *“Language is a source of hanging on to culture*, *but also a barrier to moving forward*.*”* [P0029].

Importantly, Syrian mothers needed to navigate the tension between maintaining the Arabic language in the home and knowing their children will learn English at school. The latter could be seen as a threat to maintaining the Syrian culture:

*Sometimes I feel that I’m a bad mom because I didn’t register her in preschool or any other program*. *But I’m so glad that she have learned about traditions and our community and even our tongue language*. [P0026]

*He is not allowed to speak English at home*. *So yeah*, *we’ve got like he used to go to the daycare before*. *So*, *he was in contact with only English*. *So*, *at home*, *like*, *I start to teach him Arabic*. *So forgot about English*. *As soon as you will go to school so you will learn*. [FG002]

Community level opportunities for developing language skills were viewed as facilitators for belonging and inclusion:

*Everything isn’t going as planned*. *I used to tell myself that I should finish English language in two or three years*, *and here I am completing two years in Canada*, *and I have only completed one level*. [P0026]

Lack of interpreters for some Syrian mothers proved to be barriers to healthcare access and to receiving healthcare:

*I didn’t have any interpreter*. *I took one*, *I fill the documents for the hospital*. *I feel I need interpreter for maybe I need it for something*. *But*, *uh*, *no interpreter for me*. [P0021] Participants often relied on family or friends for language support. “*My son is translating for us what ever we don’t understand*.*”* [P0013] In some cases, having family act in the role of interpreter might be a cultural risk for women, due to the Syrian stigma regarding mental illness or to the family members’ lack of knowledge about perinatal and reproductive mental health. Cultural stigma around mental illness might prevent some refugee families from accessing mainstream mental health services and supports:

*When I was to go to hospital my husband interpreter*. *He explain in English to the doctor*. *Right now its changed*. *I talk with the interpreter … and now he could understand me*. [P0035]

#### Role of gender

Refugee women’s gendered experiences may be invisible due to gender roles, education, and health literacy. In some cases, the husband’s influence was reported to be an important factor in whether the participant could seek support services. A few Syrian mothers spoke about the entrenched gender hierarchies embedded in Middle Eastern culture. Researcher field notes revealed:

The mother is very isolated in that she does not drive, and her husband does not want her to do so. He also forbids her to apply for citizenship. She spoke in a matter-of-fact way and did not appear unhappy as this is the way it is in our family. Further she said her husband wants more kids and he says she must stay home and do her job as the mother. The participant said that one day she would like to do things outside the home, and without her husband knowing. I believe a lot was unspoken. (Field notes reflections. [P0036]

Most refugee mothers interviewed had drivers’ or learners’ licences. However, through a field note it is described: *“… one Syrian mother does not go out on her own*, *nor does she drive*. *She is very dependent on her husband and acknowledges this*. *She stays home*, *as there is no option at present*.*”* Field notes (P0028)

The following comments reflect the views of several Syrian women interviewed:

*I can say that some men*, *especially from*, *like*, *our culture*, *they could say*, *okay*, *I can’t let her just go in front of me*. *I have to be in front*. *Yeah*, *they would be upset if the woman took a step in front of them*. *And they will say*, *like*, *your place in the kitchen*. *Yeah*. *So that’s our culture*. *Yeah*. *Woman only in the kitchen*. *Woman has to be in the kitchen and man*, *like*, *has to work*. *That’s the rule*. *So*, *we have to live with it*, *even Canada*. *Anywhere in the world*. [FG002]

*And the man*, *you just stay home with your kids*, *just cook*. *They’re not allowed to study because of*, *you know*, *culture*. *There’s a lot of woman here that the man doesn’t allow to study*. [P0030]

In contrast, some participants asserted that some Syrian men are open to the “less traditional role” (compared to the tradition in Syria). Even in Syria they shared gendered responsibilities and deemed the man and woman to be equal:

*I think*, *uh*, *like my husband and her husband are less traditional and they want us to drive*. *But its good to found men that are more like that*, *less traditional from back home and able to work together as a team*. [P0042]

*Someone will judge him*, *and*, *like*, *say*, *“look*, *he’s cleaning the dishes*.*” The man will be like*, *“No*, *I’m not*.*” People afraid of*, *like*, *if someone says about him*, *but the other they don’t care*, *like my husband was*, *like*, *you know*. [FG002]

Gendered responsibilities of labour in the home were clearly expressed. This participant described her friend’s accountability as described by the PRA: *“She talked about how she managed all the responsibilities (shopping*, *doctors’ appointments*, *kids meals) because of her sick husband*. *She said his condition is getting worse*. *I asked her how does she feel with this pressure because of the responsibilities*.” She answered without hesitating: “*I feel that this is my duty towards my family*.” [P0001]

Another participant in a matter-of-fact manner described the shifting gender roles in Canada compared to Syria:

*My life as a woman in Syria and Canada is totally different*. *Here*, *there are a lot of service power women to work and to be independent*, *but in Syria there are a lot of traditional beliefs that stop me to work and be independent*. *Here no difference between women and men if they have same construction job*. [Back home] *women not allowed to do men’s job at all*. *Here*, *women are free with their money but in Syria not at all*. *Such big bad words if she divorced or asks for separation*, *but here it is normal*. *If you do the grocery is normal but in Syria is going to be a big problem*. [P0026]

Keeping house was very important for this mother:

As a woman, to keep a house well is a significant part of my identity—this is a full-time duty.

She chuckled at the demographic question of being a fulltime homemaker, as no matter what her job was, this will always be a fulltime role. [P0029]

Women’s work in the home meant challenges and emotional discontent for some participants:

*Inside me I live in a state of war because I don’t have time for me*. *I am all the time busy with school*, *cooking*, *kids*.” [P006]

*Things that forbid me to work are*: *(1) my daughter*, *(2) my language*, *(3) I’m a woman*, *my husband is working*, *that is enough*, *(4) I would like to work in my field*, *medical engineering*. [P0026]

*I’ve applied for assistance but afraid about not getting work; at home women work mostly from home—middle eastern women do not have the abilities to go out because … used to staying home and looking after the family; hard to find the job*. [P0036]

One participant described the quandary as a mother of young children as translated by the PRA:

*“They cannot go to the school*, *maybe it will be some distance program for these women and because they cannot go to the school right now*, *they have two babies at home … so cannot leave them at daycare*.*”* The participant lamented: “*Its very hard for and they have to adapt so how can I improve my kids*, *my English if I don’t go to the school*. *I stay all the time at house*, *nobody can talk to me English…* [P0004]

#### Education

As noted above, many Syrian mothers aspired to improve their language skills through education in the resettlement period. Yet, some attributed their inability to attend English classes to a lack of available daycare. Participants explained:

*Its very hard to find daycare*, *its very… Some place I call them and they never replied*. *So*, *because daycare is a big issue*, *there’s lots of women that want to go to school but cannot*. [P0036]

*So how can I improve my kids*, *my English*, *if I don’t go to the school*? *I stay all the time at house and nobody can talk to me English*. *I think they need some more programs…* [P0029]

Another mother noted that financial constraints were a barrier to higher level education:

*I think*, *like*, *education here is very nice*. *It’s very hard but to get*, *to begin to get a place like in university very expensive for you*, *you need money*, *you need loans*, *you*, *uh*, *expensive and more*, *more time; long time more than our country*, *it’s very easy there*. *Education is free in my country*. [P0014]

The financial responsibility accompanying higher level education in Canada is another example of a cultural distinction that, if refugees are not informed of beforehand, might not emerge until after resettlement. One Syrian mother described a situation wherein her husband did not want their daughter to pursue further education.

[Father] *doesn’t want to take loan for her* [daughter] *and doesn’t want to send her to study; he has an old mind and doesn’t believe she needs more school*. *Doesn’t know what to do*. *She knows she’s not doing anything wrong*. *Trying to pursue getting a loan on her own without her father*. [P0035]

Aside from the financial constraints associated with higher level education, participants noted other barriers to their children’s education: “*I think its hard for refugee children … to settle into new school*. *I want to see my kids have a better way and better education*.*”* [P0029]. Another participant feared for her daughter: “*She was asked her to remove the hijab and because she thinks her schoolmate do not want to play with her just because she wears the hijab*.*”* [P0025]

Despite the challenges associated with education, one Syrian mother summarized the importance of education and the confidence, it can instill:

*I will suggest the woman*, *the Syrian woman to be confident … to be very strong*. *More confident because here in new country she has to be confident*, *she has to be strong*, *and to support her family*. *And I*, *I*, *the most important thing for me the family and the education*, *this is the most important*. [P0035]

#### Housing

After initial housing upon resettlement, participants described how overcrowding, financial constraints, and inadequate housing conditions were overwhelming stressors that impacted health, wellbeing, and safety. Participants expressed difficulties in finding adequate space for the whole family to live comfortably. Overcrowding was a recurring theme in participants’ descriptions of living conditions:

*The whole family stuck only on one bed … four—two boys and I was pregnant*. *Like my bedroom is too small*, *the back of my head is touching*, *like*, *the ceiling so*, *like*, *there’s no other*, *like*, *solution*. [FG002]

*I’m very happy but my house is pretty small for us*, *like*, *five people live in the same room and her*, *like*, *sick child in another room*. [FG001]

Whereas Syrian mothers appreciate living together with the whole family under one roof, overcrowding influences the health and well-being of all family members. However, participants who were actively looking to find more space for their families found it difficult due to family size and limited budget. In addition to paying rent, paying for utilities posed a challenge for families with one income.

### As translated by PRA

*Her husband works hard to provide a better house for them since they are six people and currently live in a small house consisting of only two bedrooms*. *She said her children are growing up quickly and they start to feel that they want some privacy that is not available in this small house*. [P0010]

*I have house problem since 2 months*. *I never show anything but now I fed up to be in this house every day*. *I look to Market place and Craigslist*. *The problem is most refuse even to meet us because we are big family*, [eight people] *as they said*. *Most of the houses are underground—sun not going inside*. *I can’t let my kids live there*. [P0006]

Aside from financial constraints and issues with neighbors, inadequate housing conditions pose difficulties. Two refugee women describe their friends’ problems:

*Especially that their neighbor in the apartment downstairs who asked them to keep quiet so she force her children to sit most of the time*. *She said that her children were so excited when he left the apartment so they could play inside without fear*. [P0038]

*She talked about how much is staying home is hard for her kids because of their new neighbor who does not allow them to play inside the house freely*. [P0001]

In contrast, one participant provided insights into her positive housing experience in Canada.

### The PRA recounted

She talked about how satisfied she is with her [housing] experience in Canada because the management of the complex she lives in decided to renew full houses even though her house still clean, but she feels that they care about the tenants and they want to give a good point of view about them. [P0005]

In a time characterized by limited affordable housing and being new to the country during this vulnerable resettlement period, refugees may be at a disadvantage in trying to secure adequate housing. This situation can have a negative influence on the health and well-being of the entire family.

#### Employment

Some mothers wanted to find a job outside the home and contribute to the financial well-being of the family. Regarding employment for themselves, participants noted difficulties in making the minimum wage and in finding suitable positions for their qualifications. Some mothers noted:

*I’m looking for a job but*, *like*, *most of the job they want*, *like*, *high level in English and sometimes they want*, *like*, *a certificate or whatever*, *like you have to have a graduate*, *like*, *something*. *I feel*, *like*, *my English is good but I told her I couldn’t find any job … but only they offer work in a factory*. [P0032]

*Look*, *I have five children*. *If I went to work*, *any work*, *I will make thirteen hour*, *fourteen hour dollar hour*. *That is not enough for this situation*. *I have to pay monthly twenty-two hundred*, *twenty-three hundred*, *just for the rent*. [P0034]

Another Syrian mother found the process of attending job interviews challenging. This mother could use more information and support.

*I really got upset*, *I have done two interview job*. *The first one was good and they accepted me*, *but personally I do not feel that is what I’m interested in*. *The second one was with xxx*. *I was so excited to get that position*, *but unfortunately the interview was too bad*. *I need to get more information how to pass the interviews in the future*. [P0002]

Consequently, some Syrian families relied on the income of the mother’s spouse. However, for some participants the financial situation is further complicated by the spouse being too unwell to work. As the PRA relayed:

*Her husband*, *who hurt his neck in a work-related accident*, *has not been working for the past four months*. *He is on a return-to-work program*, *but really cannot go back to the same heavy physical work*. *He lifted heavy windows on and off trucks since resettlement*. [P0020]

Another refugee woman shared:

*My husband works as a crane operator; usually works up in the cranes*. *He needs 3000 hours to get a full certificate to be a regular operator*, *which means he needs to set up the crane sometimes*. [He] *expressed his fear of being up in the crane*. *He hurt himself by falling from a building*, *but he had a safety harness*. *Without it he would have perished*. *He went to the hospital and is now off from work with some injury to his leg*. [P0002]

Syrian mothers noted that their role is to support their husbands: “*I can’t work because I have kids … I go to school and my husband sick*? *He needs someone to take care of him*.” [P0024]

The husband’s inability to work, and the lack of financial support, affected the health and emotional well-being of some mothers. This mother noted her struggle:

*My life gets more difficult with the current circumstances*, *as my husband is staying home after he got a work injury and too many things to worry about*. *I am very worried of our current situation as my husband is without job and our financial crisis is getting worse*, *especially with fear if we have to move to a high rent house*. [P0020]

Sending remittances to extended family in Syria or Jordan can further complicate the financial situation of Syrian refugees. One participant expressed through PRA translation:

*She is stressed about her family in Jordan and Syria*. *This is financial stress*, *as it’s very expensive in Syria—many Syrians have this problem; money is low*. *She and her husband have to send money sometimes for both their mothers*. *It’s hard to send money to both mothers and to cover their own needs*. [P0032]

### Theme 4: COVID-19 pandemic impacts and ongoing resettlement

As a result of the study’s longitudinal design (18 months) this theme emerged through participants’ written diaries and regular monthly follow-ups. As the Covid-19 pandemic progressed, some participants found it increasingly difficult to find anything new to write about in their diaries, as they were primarily staying at home. Thus, PRAs would check in with Syrian mothers and thereafter write summaries of the main conversation points. Syrian mothers testified that Covid-19 was having a substantial impact on their lives. They felt socially isolated, their access to education had been reduced and, in some cases, terminated, doctors were busy with Covid patients and their offices posed a risk of contacting the virus.

#### Social isolation

A pattern of social isolation and loneliness was evident in the Syrian mothers’ written diaries. For example, two mothers described: “*I’m crying without a reason*. *Also*, *my neighbor who I go to talk usually*, *she will be moving*, *and I will [be] alone again*. *I do not have anybody to talk to*.” [P0024] “*I try in various ways to hide any despair inside me*. *I do not want to reflect on my home and my children*. *When I smile*, *all my family smiles*.” [P008]

Many people fell back on a pre-existing social network when social distancing restrictions were first implemented due to Covid-19. But newly arrived refugees had limited access to social supports, and the COVID-19 pandemic further exacerbated their feelings of social isolation.

Several mothers worried that the lack of social contact would have negative consequences for their children. One participant said she had come to realize that she was an unsocial person and that this affected the temperament of her little son. The participant noted that her child is no longer exposed to new people, thus, when he is introduced to someone new, he starts crying; he appears to be afraid of new people. It is clear that the decrease in social support engendered by the Covid-19 pandemic had a negative affect on many refugee women and their children. Some participants were indirectly affected by Covid-19 restrictions that were placed on their families living outside of Canada. A Syrian mother wrote in her diary: *“Before*, *my mother used to support me*, *but now I don’t talk much because my father and my brother are always at home because of Covid-19*.*”* [P006]

Because the whole refugee family is constantly together during the Covid-19 pandemic, the Syrian mothers might not have spoken as openly as they would have spoken in private interviews. If refugees and their families had not established a solid social network locally within Canada, the Covid-19 pandemic worsened their chances of doing so. This would be an additional negative affect on their emotional health.

However, one Syrian mother wrote that the Covid-19 pandemic had made her spend more time with her children, and she appreciated that. It provided her with novel ways of connecting with them. This participant wrote in her diary:

*It is a great chance to sit with them* [the children] *more and understand them more and throw everything behind my back*, *any sad*, *any depression*, *any nervousness*, *because the care mother and her smile and touch are solution to all mixed feeling that may affect my children*. [P0006]

One refugee mother attempted to focus on the positive impacts of the Covid-19 pandemic in her conversation with a PRA: “She looks at the positive side of not meeting other people. She could now raise her children the way she found it appropriate, away from external influences.” [P0010]

Syrian mothers were concerned with the consequences of social isolation, both for themselves and their family. Some women managed to focus on the positive, utilizing their newfound time with their children, whereas others became increasingly worried about the wellbeing of their children. Although the social distancing implemented due to Covid-19 might be necessary also for future pandemics, these findings illuminate the importance of building social supports in normal times.

#### Changes in education

In the spring of 2020, many schools closed to limit the spread of Covid-19, and many of the refugee mothers’ children transitioned to online learning. Some Syrian mothers welcomed the idea of keeping their children at home, as it protected them from Covid-19. One mother wrote: “*I think teaching at home is better*. *It is a little difficult but it’s safer*. *I try to give my children all the education they need at home as much as possible*.” [P0028]

Other mothers were more apprehensive about home schooling. Some mothers attributed their skepticism to their own limited English language skills. After a telephone conversation with a refugee mother, a PRA noted: “She [the participant] is very mad about home schooling her kids because her English does not allow her to help them.” [P0031]

One mother believed her child would have to repeat the school year, and many participants worried that their children would not progress at the same pace as their classmates. Another participant found it difficult to structure the family’s time spent at home:

*I’m totally lost*, *I do not know what exactly I want*, *or to do*. *I feel like I have lots to do but I’m staying in my place without moving*. *I feel like I need someone help to organize my time because I think I can’t do that*. *I cannot*. [P0020]

With temporary school and daycare closures, some Syrian mothers noted that it was difficult to combine both their education and their children’s education at home. One mother wrote: “*As a level 12 student*, *there are lots of work to do*. *And as a mother of three children*, *how it would be possible for me to help them and help myself too in their and my studies*?” [P0028]

For some Syrian mothers, the closure of schools and the limited in-person social supports (such as childcare) decreased education opportunities for the whole family. School closures can also affect a family’s financial situation, exacerbating pre-existing financial challenges (loss of employment, for example). Although virtual learning was a challenge for some women, it was preferred by others. Whereas some mothers found virtual learning technically challenging and less effective than face-to-face classes, others pointed to its practicality and flexibility. Aside from education, the closure of many in-person services meant changes in access to and delivery of healthcare.

#### Changes in access to and delivery of healthcare

In the diaries, Syrian mothers noted their experiences with their family doctor during the pandemic. One mother acknowledged: *“In my opinion*, *to have a family doctor is most important thing in Canada*. *It’s a great idea but took me awhile to find one*. *Now I feel I am safe*. [P0002]

Another participant had a different point of view: “*Family doctors are so confused*, *it will be better if they put a specialist for people*, *then they go directly to visit them directly*.” [P0026]

One mother found that her mental health had become worse since dealing with her doctor during the pandemic. She felt misunderstood. Long wait times and being sent to different services complicated another mother’s healthcare experience: “*Health(care) system has not given any benefit; too long to get treated; being sent to different places is difficult*. *Felt very bad and not happy with care provided by dr*. *and hospital*.” [P0026]

Many virtual healthcare services have shortcomings that limited refugees’ access to healthcare during the Covid-19 pandemic. With many in-person services closed, participants experienced an increase in transportation time, time delays, and difficulties with scheduling virtual appointments. Long wait times and difficult access to transportation were recurring challenges for participants. One participant described: “*Appointment last week was very difficult to get to because I was so exhausted; had to take bus and train along with younger child*.” [P002]

Some Syrian mothers noted that personal protective equipment (PPE) such as masks made them uncomfortable, both in public and in the healthcare setting. One mother explained: “*Covid-19 is force us to use masks*, *put on gloves*. *This make me get scared from people*. *I don’t feel safe since these masks covers their faces*.” [P0013]

Syrian mothers also described financial challenges during the Covid-19 pandemic, primarily due to spouses’ loss of jobs. While this may have partly attributed to a change in gender roles, it also suggests that Covid-19 caused a new social inequity. We conclude that the COVID-19 pandemic had a significant negative impact on refugees’ resettlement. We suggest that in Canadian society, refugees were disproportionately affected in terms of social isolation, social inequity, shortcomings of virtual care, and a decrease in access to care.

## Discussion

This research explored Syrian mothers’ experiences and the intersecting social factors that supported their well being during their resettlement in Canada. Consistent with the Participatory Action Research (PAR) approach, we used multiple methods of data collection. We employed four Syrian mothers as peer researchers. Our analytical tool was Syrian refugee women’s narratives concerning their resettlement experiences in Canada. Specifically, we queried Syrian refugee mothers’ access to healthcare, social services, and housing. We found that Syrian mothers’ experiences were reinforced and reexperienced across migration phases. Specifically, each context of migration carried experiences of social exclusion, heightened gendered vulnerabilities, and opportunities for growth.

This research was conducted during the Covid-19 global pandemic. The pandemic added layers of complexity and heightened risks associated with the social determinants of women’s health. The global lockdown prescribed by the World Health Organization brought increased vulnerabilities to the participants of this research, affecting social support, extended networks, gendered roles, and care-taking responsibilities. While many Syrian mothers longed for home, the social advantages in Syria were also constrained by limited freedoms, by war, and by gendered cultural norms.

Factors that shaped Syrian refugee women’s migration journey remained consistent post migration to Canada. Their identities as newcomer mothers and racialized women were shaped by their often-limited social support networks, tensions related to gendered roles and responsibilities, and experiences of discrimination. However, their migration status afforded a potential for resiliency, growth, and connection. This finding is linked to hopes for their children’s future and the potential for prosperity. Consistent with other research on the effects of Covid-19 on newcomers and refugee mothers, our findings show that Syrian mothers experienced a negative impact on their health and well-being [33, 34]. These negative affects were frequently related to social isolation and an increase in their gendered, domestic care responsibilities.

In the postmigration phase, refugees faced acculturation stress, social exclusion, a decrease in community relationships, and an unpredictable access to healthcare [32, 35]. Our study focuses on the mothers that made the migration journey and suggests that mothering is not a single role or responsibility. Most Syrian mothers had extended family networks, and their mothers and mothers-in-law played vital roles in their families. This included decision making around family health and supporting new mothers in their new role as mothers. As many Syrian families did not arrive with this support, the refugee mothers were particularly challenged in their roles of mothering. This may be related to how many participants were younger in age and did not have strong role models or mentors in Canada.

Sethi and colleagues identified encouraging ways that refugee women manage the premigration trauma of violence and the post-migration isolation, racism, and poverty that impact resettlement experiences [36]. Approaches included personal efficacy, freedom of decision-making, religious beliefs, aid to family at home and abroad, a need for education, learning English, and advancement of the family. This study adds to these approaches by describing how specific Syrian cultural practices helped the Syrian refugee women to maintain their identity, and this contributed to their emotional well-being. For example, for many Syrian women, wearing the Hijab is symbolic of her identity, her culture, and her religion. Wearing the Hijab thereby facilitates Syrian women’s overall well-being. However, the frequently experienced discrimination toward wearing Hijabs were described by our participants as having a negative impact on their health.

Canadian services need to be restructured to meet the needs of newly settled refugees and their families [37]. Holistic healthcare clearly influences emotional health and physical health. Access to healthcare needs to be streamlined. Healthcare providers interact with migrants on a regular basis and serve as a pivotal point of health literacy [38, 39]. Trusting relationships with interpreters and Arabic speaking physicians with non-judgemental attitudes smoothed the bumps of refugee settlement. Physicians play critical roles in ensuring the efficient flow of health information and in supporting their clients in finding, understanding, appraising, and applying this health information. Yet, trust is essential before an effective delivery of health information can occur. A client-centered approach in the assessment of mental and physical health needs and in the delivery of health information can empower refugee women and facilitate their health literacy [38, 39].

There is need to integrate the broader context of refugee women’s daily living which ought to include social determinants of health and how they affect Syrian mother’s help-seeking behaviors. Social determinants of Syrian mothers were not dissimilar to pre-pandemic macro level determinants of integration, such as housing, labour market integration, education, and language abilities [40] Evidence shows that refugee mothers have adapted by using diverse supports [41, 42] Syrian mothers reported that their dependence on spouses for domestic responsibilities and emotional support increased in the absence of extended family networks. Our findings suggest that belonging and social support were complicated by the ongoing war in Syria and diminished when elders were left behind. Resources for social connections and the building of social relationships need to be enhanced to assist refugee settlements. Early childhood education, where the focus is often on newcomer children should be extended.

Social isolation, discrimination, hardship, and lowered economic status affect a refugee woman’s perception of control and her ability to make choices. Discrimination regarding gender, race, class, and migrant status compromises women’s socioeconomic stability and cultural integration. Marginalization and oppression impose stress and contribute to the development of depression and anxiety [22, 30]. Syrian mothers testified that the new responsibilities in the resettlement period had a significant impact on post-migration shifts in gender roles. During the settlement period, the housing conditions of Syrian refugee families displayed inadequate size, difficult accessibility, and unappealing locations. Landlord’s attitudes toward refugees were often demeaning. As the home is where assimilation begins, these factors can inhibit a smooth refugee integration into Canadian society [41].

Although times of crisis throughout history tend to exacerbate disparities for refugees, it is important to consider the existing conditions that shape the structural vulnerability of refugee women populations such as refugees [36, 37]. Structural vulnerability can be considered the social determinants of health that increase risk of poorer [42]. The COVID-19 pandemic had a substantial impact on the resettlement of refugee mothers and their families. In their diaries, refugee mothers described feeling isolated, and unequal to the task of assimilation. Access to care was limited and virtual care had its limitations. Other recent reports agree that the COVID-19 pandemic exacerbated health inequities and amplified pre-existing barriers to care for marginalized populations in Canada [42, 43]. The resources to support refugee families require urgent attention as they affect the emotional well-being and the physical health of parents, and therefore they affect parent-child relations [33, 44–46]. The refugee women in this study described feelings of social isolation and loneliness due to stay-at-home orders, limitations on in-person gatherings, and isolation requirements. Participants were stressed by having to homeschool their children and worried about their children’s limited social contacts. The refugee women’s diaries reveal that mothers were required to take on much of the family responsibility related to COVID-19. Yet, our participants showed resilience and strength-based capabilities. Stirling Cameron and colleagues also found that school and daycare closures combined with social withdrawal can be exhausting for refugee women [33].

Our work advances knowledge about mothering across multiple migration contexts. Despite encountering systemic exclusion and prejudice, Syrian mothers showed strength and resiliency. During the migration journey, many traditional social support networks for mothers and many of the gendered norms were disrupted, increasing psychological distress. However, people learn from experience and these disruptions have implications for enhancing their social support networks. Community-based organizations and non-government organizations need to increase funding for accessible programs and resources to support newcomer mothers.

This study shows that COVID-19 was a significant source of anxiety for Syrian mothers. They feared that they might become seriously ill and were reluctant to access healthcare during the peak of the COVID-19 pandemic. Refugees and migrant workers are more likely than Canadian born workers to be affected by loss of income and by health-care insecurity during a pandemic [45, 47]. Findings from this study substantiate this conclusion.

### Strengths and limitations of the study

The findings of this qualitative study cannot be generalized to all refugee women or to other geographical contexts. A larger sample and a mixed methodology with the inclusion of healthcare professionals and social service providers would broaden the scope of the research. The COVID-19 pandemic posed some limitations; for example, data could not be collected in person. But the COVID-19 pandemic also provided unplanned material for a broader investigation of the resettlement experiences of refugee mothers.

## Recommendations

### Participant endorsements

We asked the participants in the study to share their ideas about how services and support for refugee women could be improved. More support for women’s health, with programming beyond the early years of migration was a common suggestion. Examples included from pregnancy to postpartum healthcare, early menopause education, and breast cancer. It was emphasized that more newcomer groups are needed after the “early childhood years.” Other ideas included an increase social support, more language services, and childcare. Additional suggestions included education for parenting skills, more recreational facilities for women only, and accessible youth programs geared to newcomers.

### Practice

Working with the Syrian refugee women made us aware of Canadian attitudes toward and perceptions about refugees. Discriminatory attitudes and behaviours marginalize women and negatively impact their sense of self. To decrease discrimination against refugees, healthcare providers must engage in advocacy practices and speak up about discriminatory practices and policies within Canadian organizations. A critical element of social justice is advocacy also includes fostering welcoming communities across service sectors such as settlement services. Cultural safety and humility can enlighten healthcare providers regarding the complex social and cultural realities that impact refugee women’s daily lives, and therefore their health. On-going healthcare training needs to recognise the discriminatory behaviour women often experience. Strategies to help women deal with negative attitudes and racism should be an integral part of healthcare training. Healthcare providers can help women to recognize they are experts on their own lives. It is imperative that women be given the ethically, legally, and morally appropriate tools to speak and be heard. Translation and interpretation services in healthcare interactions can help refugee women to find their voices. Language diversity in health information available to clients continues to be of critical importance for men as well as women. Women might receive inaccurate information from their partners. The successful resettlement of refugees requires cross-sector partnerships of local community resettlement organizations, educators, refugee serving agencies, and healthcare providers.

### Policy

Healthcare providers are in an optimal position to advocate for the rights of refugees in healthcare institutions, in health professions, and within the community. Building public policy that supports Syrian mothers’ mental health and physical well-being is subject to the unequal distribution of Canada’s wealth and its opportunities for education. As refugee women’s health is affected by the social determinants of health in Canada, we have a moral obligation to address how gender, health literacy, migrant status, and education intersect, and to restructure funding to address this intersection [48].

Funding affordable housing projects and developing rent control policies can support the financial stability of refugee women. Many women were keen to continue their education in Canada, but perceived language and cost to be barriers. The unavailability of childcare services challenged women’s obtainment of language skills, education, and adequate employment. Funding for refugee education and re-training programs can add skilled workers to the workforce. Policies that facilitate extended family migrations are needed. Although policies that support family reunification in Canada do exist, government targets limit the number of yearly applications and thereby exacerbate delays in reunification processes [49]. Policy makers who support refugee women’s resettlement need to analyze how government target decisions are impacting family reunification policies. Such analyses can facilitate identification of strategies to enhance flexibility and mitigate barriers embedded within government target decision-making. Prolonged family separation contributed to stress in the resettlement of refugee women.

Local Immigration Partnerships (LIPs) involves service providers, settlement agencies, community groups, and employers in welcoming newcomers to Canada [50]. A strong presence of LIPs in communities where a diverse representation of migrant statuses, races, and genders are included is recommended to develop and implement frameworks for improving the settlement and integration of newcomers. LIPs provide opportunities for immigrant women to network with other newcomers, to learn about cultural differences in Canada, to access language, to connect with health systems, and to obtain housing support.

### Research

Researchers invested in studying the social well-being, the physical health, and the mental health of Syrian refugee women need to consider larger, mixed methods studies to inform program and policy development. Non-traditional and creative qualitative research methods, such as photovoice, poetry, and sharing circles also enable refugee women to engage in storytelling as a form of resistance against oppressive systems and structures. Research exploring the experiences of refugee women living in rural communities could reveal differences in the level and kinds of social support available to rural refugees.

## Conclusion

Four specific findings in this study add to current understandings of refugee women’s health and well-being. (1) Syrian refugee mothers face social exclusion, uncertain community relationships, and diminished access to healthcare, especially when they are not connected to settlement services. (2) Syrian refugee mothers continue cultural and religious practices to draw strength from after resettlement. Specifically, addressing discrimination enhanced overall well-being. (3) Social and health services need to be restructured to build trusting relationships between service providers and refugee women. Specific attention to affordable housing would address the disproportionate inequities faced by refugee women in their settlement journeys. (4) The COVID-19 pandemic in Canada caused significant setbacks in refugee women’s settlement trajectories.

To honour and validate refugee voices that have been silenced in the past, there is a moral responsibility to hear the voices of these participants. Through these research findings we can improve social justice. The testimonials of refugee women expand our understanding about how social determinants can impact mental health and emotional well-being. By exploring social factors such as economic insecurity, care giving, family responsibilities, and resettlement experiences, we see how they influence a refugee women’s ability to access and utilize appropriate services. The ability of refugee women to utilize appropriate services ultimately affects their health, the health of their families, and the ability of these families to function productively in Canadian society.

The longitudinal findings obtained in this study can provide support services that are understandable, accessible, and culturally appropriate for refugee women living in BC. Knowledge gained from this research increases the awareness of challenges Syrian mothers face in resettlement.

## Supporting information

S1 Appendix Demographic table. (DOCX)

S2 Appendix Interview guide-questions. (DOCX)
