# Supplementary material for: Use of participatory action research to support Syrian refugee mothers in the resettlement period in Canada: A longitudinal study
Source: PLoS One. 2023 Feb 21;18(2):e0281765. doi: 10.1371/journal.pone.0281765 (PMC9942982; doi:10.1371/journal.pone.0281765)
Supplement: S1 Appendix — (DOCX) [file pone.0281765.s001.docx]

**S1: Sociodemographic Table**

| **Sociodemographic Table** | N = 40 |
| --- | --- |
| **Age** |  |
| - 21 – 30 | 13 (32.5) |
| - 31 - 40 | 22 (55) |
| - 41 - 50 | 4 (10) |
| - 51 or higher | 1 (2.5) |
| **Marital status** |  |
| - Married | 36 (90) |
| - Divorce/Separated/Widowed | 4 (10) |
| **Children** |  |
| - 1 – 2 | 4 (10) |
| - 3 – 4 | 24 (60) |
| - 5 – 6 | 10 (25) |
| - Participant demographics | 2 (5) |
| **Years in Canada** |  |
| - < 1 | 1 (2.5) |
| - 1 – 2 | 7 (17.5) |
| - 3 – 4 | 31 (77.5) |
| - 5 or more | 1 (2.5) |
| **Sponsorship type** |  |
| - Government assisted refugee | 36 (90) |
| - Privately sponsored refugee | 4 (10) |
| **First Language** |  |
| - Arabic | 38 (95) |
| - Kurdish | 2 (5) |
| **Education** |  |
| - University/College graduate | 7 (17.5) |
| - Some university or college | 1 (2.5) |
| - High school graduate | 8 (20) |
| - Some high school education | 8 (20) |
| - Less than high school education | 15 (37.5) |
| **Income** |  |
| - Less than $10,000 | 2 (5) |
| - $11,000-$20,000 | 12 (30) |
| - $21,000-$30,000 | 12 (30) |
| - $31,000-$40,000 | 0 (0) |
| - $41,000-$50,000 | 2 (5) |
