# Supplementary material for: Use of participatory action research to support Syrian refugee mothers in the resettlement period in Canada: A longitudinal study
Source: PLoS One. 2023 Feb 21;18(2):e0281765. doi: 10.1371/journal.pone.0281765 (PMC9942982; doi:10.1371/journal.pone.0281765)
Supplement: S2 Appendix — (DOCX) [file pone.0281765.s002.docx]

S2 Interview Guide/Questions: (Created by Syrian Peer Researcher Assistants Research Team Members)

Tell me a little bit about how you came to Canada?

- How long have you been here?

OR… I would like to start with when you arrived to Canada – how was the first day? And

how did it feel? When you arrived what was the most difficult thing?

What was your life like before you arrived?

- How did it feel to live in Syria? What was the most difficult part of leaving your home country?
- Do you have family still living back home?

Do you have family or friends here in Canada? Living close by?

- What are things you expected to find and you didn't find it?
- As a mother how did you feel looking after your children without having extended family support?
- What family support was provided when you first settled?

Can you tell me about your present health?

- Is there anything you would like to tell me about your health care experiences? …tell me a bit about what it was like getting healthcare for you and your children (Prompt-when you had to see a doctor or nurse)

Do you or have you ever felt down or sad during your first years in Canada?

- Is there ever a time where you felt physically or emotionally unwell after the birth of your baby?
- Some women experience feeling tired, low energy and unable to mage life, sometimes feel sad...has this ever happened to you? What was that like? What supports were needed but you could not find?

Have you had to access special health care services for extra support for your emotional well-being? OR if you felt like you needed more support, who would you go to?

- Has being a woman influenced how you have managed your health? For example, are you comfortable with male/female doctors?
- If the help was formal/professional who would that be? health care provider? nurse? doctor? Many women access midwives in the community, is that something you are familiar with?

What is family support like for you in Canada? How does the support help your wellbeing and health?

- Is there anyone that you can talk to if you feel bad or stressed? Do you think that you would like to talk to somebody from your own culture about health?
- What kinds of cultural beliefs and values influence the ways you self-manage your health?

What do see as your strengths, in your family, and community? Can you suggest something that is very important to you that health care providers and other services should know about your community?

What do you think prevents other newcomer women from getting help for their emotional health? What would help?

Is there anything else you would like to share today before we close...
